# Anthrax Toxin Detection: From In Vivo Studies to Diagnostic Applications

**DOI:** 10.3390/microorganisms8081103

**Published:** 2020-07-23

**Authors:** Jean-Nicolas Tournier, Clémence Rougeaux

**Affiliations:** 1 Unité Bactériologie Biothérapies Anti-infectieuses et Immunité, Institut de Recherche Biomédicale des Armées (IRBA), 1 place Général Valérie André, 91220 Brétigny sur Orge, France; jean-nicolas.tournier@intradef.gouv.fr; 2 Centre National de Référence-Laboratoire Expert Charbon, 1 place Général Valérie André, 91220 Brétigny sur Orge, France; 3 Innovative Vaccine Laboratory, Institut Pasteur, 28 rue du docteur Roux, 75015 Paris, France; 4 Ecole du Val-de-Grâce, 1 place Alphonse Laveran, 75005 Paris, France

**Keywords:** anthrax, toxins, lethal factor, edema factor, protective antigen, ELISA, mass spectrometry

## Abstract

Anthrax toxins are produced by *Bacillus anthracis* throughout infection and shape the physiopathogenesis of the disease. They are produced in low quantities but are highly efficient. They have thus been long ignored, but recent biochemical methods have improved our knowledge in animal models. This article reviews the various methods that have been used and how they could be applied to clinical diagnosis.

## 1. Introduction

*Bacillus anthracis* is a Gram-positive spore-forming bacterium, considered one of the most potent and critical bioterrorist agents, and subsequently is listed as Category A select agents by the Centers for Disease Control and Prevention (CDC). It is responsible for anthrax, a zoonotic disease, mainly affecting herbivores, humans being only occasional hosts. There are three main forms of human anthrax and a recently described atypical form, depending on the route of entry of the pathogen: cutaneous, gastrointestinal, inhalational, and injectional anthrax.

*B. anthracis* toxins largely shape the pathogenesis of the disease in mammals, even though these proteins are produced at very low levels. Indeed, anthrax toxins are highly efficient, as most of their effects are biochemically amplified. Thus, their detection is very challenging, as the toxins are present in the blood at very low levels, below classical detection methods, and are not accessible to the genetic amplification methods used for molecular diagnosis. 

However, recent studies have proposed new methods for the detection of anthrax toxin, leading to reassessment of the pathogenesis of anthrax through the lens of the toxins, and leading to exciting perspectives for anthrax diagnosis. *B. anthracis* needs having sensitive, rapid, and scalable methods of detection of the organisms as of its toxins.

## 2. Why Detect Anthrax Toxins? 

Anthrax toxins act at two critical stages of the infection [1]. Early in the infection, they paralyze the immune response of the host by targeting innate and adaptative immune cells. During the late stage of the infection, the toxins are involved in the failure of vital organs by acting on target cells.

An uncharacteristic clinical picture, with the exception of the cutaneous form, and first-line antibiotic treatment can complicate the initial diagnosis of anthrax. It is currently based on bacterial isolation in cultures and the detection of specific markers of *B. anthracis*, as antigens or nucleic acid using *pagA* PCR and, more recently, detection of BA_5345, a chromosomal marker allowing differentiation between *B. anthracis*, *B. cereus* biovar *anthracis*, and *B. thuringiensis* [2,3,4]. However, these diagnostic approaches have their limits, as bacterial clearance due to early antibiotherapy and the low sensitivity of the test and time required to perform them are not compatible with rapid management of the disease. Sensitive and rapid assays for the detection of *B. anthracis* are needed to facilitate early and accurate diagnosis and post-exposure treatment. During a bioterrorist attack, for example, the screening must be rapid and should allow the testing of a large number of samples.

Many technical approaches for the detection, identification, and quantification of the toxins of *B. anthracis* have been developed and sometimes used in the laboratory.

## 3. How Does One Detect Anthrax Toxins and for What Applications? 

Anthrax toxins are formed by the association of three proteins that are individually non-toxic—the protective antigen (PA), the lethal factor (LF), and the edema factor (EF). PA plays a role in the cellular binding and entry of the toxins. After secretion by *B. anthracis*, PA83 binds to its cellular receptors: ANTXR1 or tumor endothelial marker-8 (TEM-8), ANTXR2 or capillary morphogenesis protein-2 (CMG-2), the two majors receptors identified, and, to a lesser extent, LDL receptor protein-6 (LRP-6) and integrin β1 [5,6]. After binding, PA83 is cleaved by furin proteases [7] into PA20, which is released, and PA63, which forms an oligomeric structure allowing the binding of EF and or LF [8], thus forming edema toxin (ET) and or lethal toxin (LT).

LF is a zinc-dependent metalloproteinase that cleaves and inactivates the mitogen-activated protein kinase kinases (MAPKKs) 1–4, 6, and 7 [9]. It has been recently shown that LF can also cleave the NLRP1b (nucleotide-binding domain leucine-reach repeat protein) of some susceptible rodents, constitutively activating the inflammasome and leading to cell death by pyroptosis [10,11].

EF is an adenylyl cyclase converting ATP in cAMP in the cytoplasm of eukaryotic cells [12]. In turn, the important increases in cAMP concentrations activate two main factors—protein kinase A (PKA) and the exchange protein activated by cAMP (Epac) [13,14].

The detection of the toxins of *B. anthracis* to diagnose anthrax has been used for decades, since their discovery and identification in the 1950s [15,16,17,18,19]. Several methods have been developed, with varying sensitivity and specificity—many first focusing on the protective antigen (PA) and then on the lethal factor (LF) and edema factor (EF).

### 3.1. Directly: The First Approach to Detect Anthrax Toxins

An agar-diffusion method based on the technique of Ouchterlony was developed in 1957 to titrate PA [20] (Table 1). This method enabled the differentiation of the three factors constituting the toxins of *B. anthracis*, the titration of each correlating with their lethal capacity [19]. The Ouchterlony method consists of precipitating the sought antigens with known antibodies on gels and was used for a long time to determine the presence of toxins in vitro and in vivo [21,22], with an application for a serological survey after an outbreak of human anthrax in the USA [23,24,25,26]. The kinetics of toxin production have also been studied using the Ouchterlony method in guinea pigs and rhesus monkeys challenged with spores of *B. anthracis* [27]. The toxins appeared to be present in the thoracic and peritoneal fluid of all dead guinea pigs, death occurring within 18–42 h, depending on the strain. During the infection process, the earliest time of detection was 6 h. In monkeys, the toxin was detected in the blood 16 h after challenge, as were the bacteria.

This technique enabled direct detection of the toxins but was less sensitive than the indirect hemagglutination test (IHA) and enzyme-linked immunosorbent assay (ELISA) methods developed late, and provided more qualitative than quantitative information (Table 1).

### 3.2. Antigen Detection

Mabry et al. [29] developed an ELISA to detect PA and LF, notably in the serum. ELISA allowed the detection of PA in the late stage of infection in guinea pigs intranasally challenged with a strain of *B. anthracis* or after death in a rabbit inhalation model of anthrax. The authors explained the absence of detection of PA or LF at the early stage of infection by the fact that anthrax toxin released into the circulation continuously binds to the available tissue receptors until saturation. However, further studies using more sensitive methods showed this not to be true.

Another technique—western-blotting—allowed late quantification of LF, EF, and PA 48 h after infection in a rabbit model of cutaneous anthrax infection [32].

Several time-resolved fluorescence (TRF) immunoassays were developed to detect PA. Thus, a specific, sensitive, and rapid europium nanoparticle-based immunoassay (ENIA) has been studied for the detection of PA [33]. The immunosorbent assay format was adapted with the use of fluorescent europium nanoparticles (Eu^+^ NPs), a nanoparticle with a large surface area and, consequently, the capacity to bind a very large number of molecules. An anti-PA antibody able to bind PA83 and PA63 was used. The immune complex was then coupled to streptavidin-coated Eu^+^ NPs and fluorescence was measured. However, the assay, conducted in PBS and animal plasma, was mainly qualitative or semi-quantitative.

Another TRF immunoassay allowed detection of PA (PA83 and PA63 associated with LF) in human sera from acute (Table 4) and convalescent patients [36], with a variable limit of detection (LOD). PA was detected in nine of 10 confirmed cases of patients with cutaneous, inhalation, and gastrointestinal anthrax 1–11 days after onset of the disease. In the patient that did not present PA, anti-PA IgG was detected. The therapeutic anti-PA IgG antibody likely resulted in a large decrease in PA, below limit of signal detection of just over 1 µM. LF was also detected in nine of these patients, LF levels being mostly lower than those of PA [40]; the lowest level of LF was 0.035 ng/mL for a cutaneous anthrax case and the highest, 57.98 ng/mL for an inhalation anthrax patient.

Other assays have been assessed without a true application (Table 1). Hence, tests based on energy-transfer, such as amplified luminescent proximity homogeneous assay (AlphaLISA) [37] and homogenous time-resolved fluorescence (HTRF) [38], were developed, enabling the detection of PA in the sera of anthrax-infected rabbits, with a low detection threshold and rapid obtention of the results. The presence of PA in serum samples has also been detected using an electro-chemiluminescent immunoassay (ECLI) in rabbits [30] and in African green monkeys [31], surface plasmon resonance (SPR) technology [35], and a metal-enhanced fluorescence (MEF) assay [34].

An alternative method using mass spectrometry (MS) was recently established. PA83 and PA63 were immunopurified using magnetic beads covered with two anti-PA monoclonal antibodies (mAbs) and hydrolyzed by trypsin [39]. The specific tryptic peptides were analyzed using LC-MS/MS, with low detection limits for plasma, allowing the detection and quantification of total PA (PA83 + PA63) and PA83.

These techniques all present the following advantages: ease of use, a low detection threshold, and a rapid test for some (Table 1). However, they do not indicate the functionality of the toxins.

### 3.3. The Enzymatic Activity of LF and EF as New Targets of Detection and Applications

A method to detect protease activity of the lethal toxin (LT) has been recently reported, combining the high sensitivity of PCR with the ability to detect the endopeptidase activity of the bacterial factor [41] (Table 2). Briefly, LF was captured using PA63 heptamers. This complex was added to a peptide-DNA conjugate, the peptide being specific to LF [42]. The cleaved DNA was released into solution and amplified by real-time PCR. This method allowed the detection of 10 fg of LF spiked into HEPES and 50 fg spiked into human serum. Another method was based on the detection of a fluorogenic peptide substrate mimicking MAPKKs in the plasma [43]. After the capture of LF and exposition to the peptide, the enzymatic activity was determined either by HPLC or a microplate reader. The limit of detection was less than 5 pg/mL after 2 h using HPLC and 20 pg/mL in 5 h using a microplate reader. Without the capture of LF, direct monitoring of the enzymatic activity of LF in the sample showed a limit of detection of <1 ng/mL to 25 ng/mL in 5 h and 15 min, respectively. However, these promising methods have not been tested on clinical samples.

For EF, the depletion of ATP was monitored by inhibition of a luciferase-mediated light-emitting reaction [48]. However, to ensure that the depletion of ATP is due to EF, anti-EF antibodies had to be included in the assay. In this way, the assay could be applied for the evaluation of the anti-EF humoral response in experimental animals infected and/or vaccinated with/against *B. anthracis*.

Another sensitive enzymatic assay relied on increasing the level of cAMP to detect functional EF [47]; the production of cAMP by the EF adenylyl cyclase was monitored in the presence of calmodulin and calcium, using a competitive immunoassay, directly in a matrix. Thus, EF could be detected at concentrations of 1 pg/mL in human plasma in 4 h and 10 pg/mL in the plasma of various animals. This method was applied to study the kinetics of production of EF during cutaneous anthrax in a mouse model of infection [46], allowing rapid and sensitive detection of EF early in the infection at the initial site of inoculation and in the blood.

A team at the CDC has established a specific and sensitive method using MS for detecting LF activity in serum in less than 4 h, allowing its integration into the CDC response plan during an anthrax emergency [42]. Total LF (LF and LT) was first purified and concentrated during an immunocapture step. The captured LF was then exposed to a specific peptide substrate mimicking MAPKKs. The two peptides, produced by their cleavage by LF, were analyzed by MALDI-TOF MS. PA did not interfere with the immunocapture step or the cleavage reactions. The LOD varied depending on the volume of the sample analyzed: 0.005–0.25 ng/mL for 200 to 5 μL, respectively. An extended reaction time also improved the detection limit. This method was applied in a model of inhalation anthrax in Rhesus macaques (RMs) with the detection of LF at the late stage of infection [42].

LC-ESI-MS/MS gave similar results (detection limits, accuracy, and precision) but analysis took longer [44]. EF activity can also be detected via its adenylyl cyclase activity and the production of cAMP by LC-ESI-MS/MS.

Over the years, LF and EF detection have been refined, providing lower detection thresholds (Table 2) and thus the earlier screening of anthrax.

As LT is the form that acts on cells and tissues, the following three-step method to detect and measure the LT complex was developed [45]: magnetic immunopurification used an anti-PA monoclonal antibody (mAb) (capture of free PA and LT), captured LT cleaved a MAPKK-like peptide, and the products of cleavage were detected and quantified by MALDI-TOF MS. The longer incubation time allowed confirmation of low-level positives and the ruling out of negatives, with a specificity of 100%. This new development enabled the differentiation of total LF and LF associated with PA (LT). The same principle was applied for PA and EF, allowing the detection of total EF vs. ET (EF associated with PA, edema toxin) and total PA vs. PA83 in rabbits and RM during inhalational anthrax [49,50]. Total EF (EF + ET) was concentrated after an immunocapture step using one EF mAb and two PA mAbs [44,51]. Concentrated EF then cleaved ATP into cAMP, which was detected using LC-MS/MS, with a detection limit of only 20 fg/mL.

**Figure 1:**
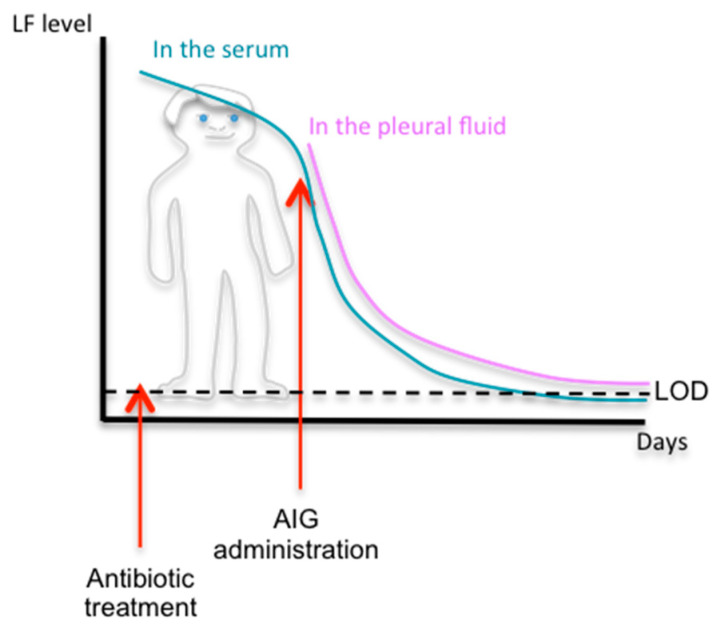
Evolution of LF levels during inhalation anthrax in human, adapted from references [52,53].

These methods were applied for various cases of human anthrax, focusing on the detection of LF. In a patient with inhalational anthrax, LF protein was detected several days after the apparition of symptoms (Table 3) and the initiation of antibiotic therapy in serum, plasma, and pleural fluid samples using a quantitative MS technique (see Figure 1), showing that the toxins are not cleared after antimicrobial therapy and that LF remains detectable in the blood for 12 days [52]. Concentrations varied between 200 ng/mL and 543 ng/mL depending on the fluid analyzed (543.2 ng/mL in the early pleural fluid sample). LF Levels were determined during therapy, making it possible to follow the effect of the antibiotics: LF levels in the serum-plasma and pleural fluid decreased steadily, with a marked diminution of LF in the plasma to 0.85 ng/mL 1 h after the administration of anthrax immune globulin (AIG). For another case of human inhalational anthrax, LF levels were assessed in serum samples and pleural fluid by MS [53] (Table 4). The initial level of LF in the plasma was 58 ng/mL, decreasing to 1.5 ng/mL after AIG completion; the concentration of LF in the pleural fluid was 16.2 ng/mL at initial drainage, decreasing during treatment.

After an outbreak of cutaneous anthrax in Bangladesh, LF toxemia was quantified by MS [40]. LF was present in acute serum (day 3 to day 8), with levels from 0.005 ng/mL to 1.264 ng/mL for 69% of individuals. LF was not detected in convalescent serum (day 16 to day 28), confirming the efficacy of the treatment. The same profile was observed for a patient with anthrax-like eschar [56]; LF was detected in the acute plasma but not in the convalescent samples of the patient.

A two-step technique based on LC-MS/MS without an immunocapture step was used in a mouse model of cutaneous anthrax to understand the kinetics of LF during infection [46] and in a mouse model of inhalation anthrax [57]. Although the detection limit was higher than that of the three-step method, it allowed the rapid detection of LF at the early stage of infection.

## 4. Toxins In Vivo

For a long time, little was known about the physiological level of toxins produced by *B. anthracis* during infection. The main hurdles were the low quantity of the toxins produced and the limited tools available to measure them. Recent developments in biochemistery, with more sensitive techniques, have provided a more precise picture of what happens in vivo during (i) cutaneous anthrax, the most common form, and (ii) inhalation anthrax, the most fulminant and deadliest form.

### 4.1. Cutaneous Anthrax

Dal Molin et al. were the first to quantify the level of PA, LF, and EF during cutaneous anthrax in a rabbit model [32]. Blood samples were collected every 24 h and bacterial factors quantified by western blotting. They were not detected 24 h after infection, but at 48 h, PA63, LF, and EF were detected, whereas PA83 was never observed (Table 3). The LF/EF ratio of ≈ 5 remained relatively constant.

Detection may depend on the bacterial load. When mice were subcutaneously infected with 10^3^ spores of the Sterne strain, PA was never detected (from 6 h to 237 h) [33]. However, when mice were challenged with 10^7^ spores, PA was first detected at 24 h post-infection at a concentration of approximately 68 ng/mL, when the rodents started to present symptoms of the disease. When the mice were ill at 42 h and 48 h, PA concentrations increased to 408 ng/mL. At 6 h and 8 h, PA was not detected in the still healthy mice.

These techniques are not sufficiently sensitive and did not allow observation of what happened earlier during the infection process. However, they correlated the presence of PA in the blood with an advanced state of the disease. The development of MS enabled a more rigorous vision during the course of infection.

In a mouse model of subcutaneous anthrax, LF was quantified 12 h after challenge, in the ear, the draining cervical lymph nodes (cLN), and serum by MALDI-TOF MS [54] (Table 3). This early presence reinforces the dogma of the paralyzing effect of LT on PMNs, as demonstrated in vitro on cells and in vivo by injection of LT, thus protecting the bacteria from the immune system [55].

A further study defined three stages of infection, depending on the location of bacteria—early, mid, and late. At each stage, LF was quantified in several organs [55]. When the bacilli were detected in the inoculated ear (early stage), LF was detected in many tissues—the infected ear, serum, cLN, heart, lungs, spleen, and liver, but not the brain or bone marrow. LF concentrations then increased during infection, and LF was detected in all tissues analyzed. The authors noted that LF levels at the infection site were higher than those observed in the serum and bone marrow during the early and mid-stages of infection, suggesting that LF found at the site of infection may play a greater role in initial survival and escape from the innate immune response than that of circulating LF.

**Figure 2:**
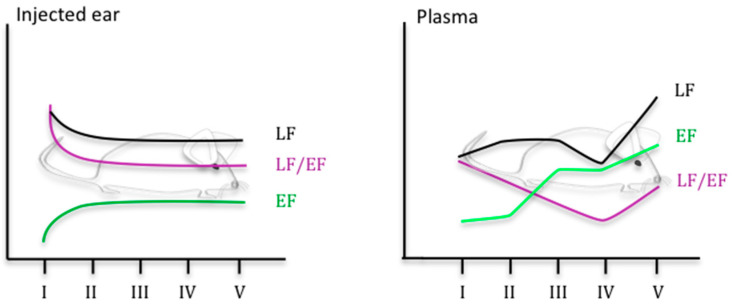
Kinetics of LF and EF level in a mouse model of cutaneous anthrax, adapted from [46] (stage I to V defined through BLI imaging. Stage I: no BLI, stage II: BLI in the injected ear; stage III: BLI in the injected ear and in the draining cLNs; BLI in the injected ear, in the draining cLNs and in the spleen; stage V: mice in septicemia).

More recently, the use of LC-MS/MS and EIA assay has provided a picture of the complex kinetics of LF and EF in a mouse model of cutaneous infection [46] (see Figure 2). Thirty minutes to 3.5 h after infection with spores of *B. anthracis*, LF and EF were detected in the site of inoculation (ear) (Table 3), in accordance with a rapid germination of spores and a rapid toxin production. More surprisingly, despite the absence of circulating bacteria, LF and EF were also detected in the blood (Table 3). Although only 29% and 38% of the mice were positive for LF and EF at the site of infection, respectively, the percentage increased to 94% positive mice for EF and/or LF. The percentage in the blood was lower (62% positive mice) when detection of the two was combined, LF being the more effective blood marker of disease. In the ear, the percentage of mice positive for EF and LF increased during infection, with an associated decreased level of LF and an increased concentration of EF. The measured LF/EF ratio varied between 320,000 at the early stage of infection and 890 at the terminal phase. As described in the study of Weiner et al. [55], the level of LF was higher at the site of infection than in the blood until the stage with a bioluminescent spleen. LF and EF concentrations in the blood tended to increase during infection, with a slight decrease of LF at the stage of infection preceding the terminal phase. The LF/EF ratio was 3 just before the terminal stage of infection, corresponding to the previous value of 5 determined by Dal Molin et al. in their rabbit model of infection [32] and approaching the values observed in an inhalation model of anthrax in RMs [51].

Cutaneous anthrax is mainly a local form that leaves a black scar. Patients generally recover without treatment, but in some cases, the infection can spread and kill. The data primarily collected in animal models indicate a more diffuse infection, with virulence factors detected in the blood explaining the rarely fatal outcome of this form.

### 4.2. Inhalation Anthrax

In the guinea pig model of inhalation anthrax, PA was detected in sera by ELISA just before or just after the death of the animals for four of five infected animals [29] (Table 4). PA and LF were detected in two infected rabbits after their death. Improvements in techniques have allowed faster detection of PA in these animal models [30] (Table 4). ECLI allowed the detection of PA in 44.4% of rabbits 18 h after challenge, whereas ELISA allowed the detection of PA in only 11.1% of rabbits 24 h post-infection (both, however, prior to bacterial detection). The discrepancy in the time of detection is explained by the difference in the LOD between ELISA (10 ng/mL) and ECLI (1 ng/mL). The PA concentration increases over time, similar to the increase of bacteremia, with a final concentration that can reach 5 μg/mL. In guinea pigs infected with various doses of Vollum spores, PA was first detected at 24 h in 10% of animals and all bacteremic animals showed detectable PA from 30 h post-challenge, with a maximal concentration of ≈ 40 μg/mL at the final stage of infection.

After focusing on PA detection, techniques were also developed to detect LF and EF.

**Figure 3:**
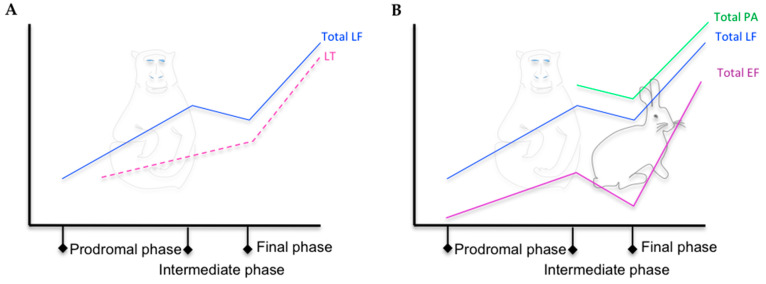
(**A**) Kinetic trends of total LF and LT level in the serum of RMs with inhalation anthrax, adapted from [45]. (**B**) Kinetic trends of total LF, EF, and PA level in the serum of RMs and New Zealand white rabbits with inhalation anthrax, adapted from [39,45,50,51,58].

The MS method was first used in a model of inhalation anthrax in RMs, in which LF was detected in the serum of all three RMs infected at a concentration of 30 ng/mL to 250 ng/mL two days after infection and 30 to ≈ 550 ng/mL the day of the animals’ death (2–4 days) [42]. MS and ELISA were then used in the same animal model for LF and PA detection, respectively [58] (Table 4). These techniques were compared to classical diagnostic tools for anthrax, which detect the *pagA* gene by PCR. It allowed the observation of a triphasic kinetic profile (Figure 3B) for LF in the serum of four of the five animals tested: LF was detected in three RMs 24 h after infection (60% of positive RMs), more rapidly than in the first study [42], at levels ranging from 0.006 ng/mL to 0.2 ng/mL. The LF concentrations were higher at 48 h and then decreased by 72 h. By 96 h, the LF levels were increasing for three of the animals, whereas they continued to decrease for the other two. At 120 h, the LF concentration was increasing for all animals. PA was detected only at 96 h and 120 h, the levels of samples for time points earlier than 96 h being lower than the detection limit of 4.8 ng/mL. At the late stages of infection, PA levels were higher than LF levels. The PCR of *pagA* was positive for four RMs by 48 and 72 h. The PCR for *pagA* reverted to negative at 72 h for one animal, which showed the lowest LF levels, suggesting microbial clearance. These data suggest that early during infection, either more LF is produced or it is less rapidly sequestered by the host tissues than PA; the circulating level of PA is sufficient to potentiate early infection and anthrax bacteremia. In the same animals, EF was first detected in the serum of two RMs (0.16 pg/mL and 0.42 pg/mL, 40% of positive RM) at 24 h post-challenge and in the serum of the three others at 48 h [51]. The detection of both LF and EF at 24 h post-challenge resulted in 80% positive animals. EF remained detectable throughout infection, with a maximal level of 2220 ng/mL. For the RM that died, the LF/EF ratios ranged from 3.6 to 17.5. The study of Solano et al. completed this kinetic analysis by focusing on the detection of PA83 and PA63 in the same five RMs [39]. PA63 was first detected 48 h after challenge in all RMs, at the intermediate phase of the disease (Figure 3B), at higher levels than LF. Such an excess of circulating active PA could constitute a reservoir for toxin formation throughout the infection. The continuous hydrolysis of PA83 to PA63 may explain the transient presence of PA83 at lower levels and the absence of its detection during cutaneous anthrax, although the technique used was less sensitive [32].

In rabbits, all animals exposed to various doses of Ames spores that developed anthrax had detectable toxins [50]. LF was first detected at 12 h and EF and PA were detected later (Figure 3B). The level of PA was higher than that of LF and EF. As observed in RMs [39,45,51,58], EF concentrations tended to match those of LF and PA at the final phase of infection (Figure 3B). Also as observed in RMs [39], PA63 predominated, PA83 being detected only punctually.

Contrary to the macaque model, intranasally challenged mice showed detectable LF in the plasma of all animals 1 h after challenge, at a mean concentration of 2.63 ng/mL [57]. However, as for RMs, EF was detected in only ≈ 42% of infected animals in the early phase of disease at much lower levels than LF.

Boyer et al. focused on the two forms of LF—free LF and LT, which were quantified in the serum of two RMs during aerosol-inhalation anthrax [45] (Figure 3A). Free LF was first detected at 18 h in the first macaque at a level of 0.026 ng/mL and 24 h post-exposure in the second macaque, before the detection of LT, bacteremia, or *pagA* by PCR. Both animals were positive for LF, LT, *pagA* PCR, and bacteremia at 36 post-exposure, the level of LF level being higher than that of LT. The triphasic profile observed in previous and subsequent studies was found in this study for total LF (see Figure 3). This analysis demonstrated a majority of free LF in the earliest stages of infection and a dominant LT form at the late stage, with LT representing 100% and 60% of the total LF for the two animals.

## 5. Conclusions

Human anthrax is a rare disease, but endemic/enzootic foci persist, and there is an ever-present bioterrorist risk. It its therefore important to have sensitive and ultra-rapid techniques for early diagnosis of the disease. The sooner the patient is diagnosed, the more effective the treatment administered and the better his chances of survival, especially in cases of inhalation anthrax.

This review highlights very significant technical progress that has made it possible to better understand the mechanism of infection of *B. anthracis*, with the kinetics of toxin diffusion challenging certain dogmas. In the future, these techniques may constitute very promising diagnostic tools for the laboratories that do not use them yet routinely.

The studies cited in this review show that LF, PA63, and EF are secreted very early after infection and that they rapidly diffuse and circulate very in the blood. LF has been shown to reach certain tissues just as quickly, playing its deleterious role on the immune system. It is likely that the same is true for EF and PA. The triphasic profile observed for their concentration during infection in RMs, rabbits, and mice is consistent with the profile of the course of the disease. Their level increases relatively quickly during the prodomal phase, then reaches a plateau or slightly decreases during the intermediate phase, and then increases markedly during the terminal phase. Thus, in the fulminant and usually fatal form of anthrax, early symptoms are non-specific (corresponding to the prodromal and intermediate phase), followed by “stormy” deterioration of the patient’s state, with multi-organ failure (terminal phase of the disease). This implies strong and rapid aggression of the host by *B. anthracis*, which explains the faster and greater immune response than that observed for cutaneous anthrax.

The ability to detect the toxins provides several advantages. (1) As their levels increase quickly, they can be detected early, especially when there is a suspicion of anthrax, before any clinical signs, which is very important, as the initiation of adapted anthrax therapy during the prodromal phase significantly improves survival [59]. Moreover, searching for LF and EF increases the chances of detection. (2) Their detection is independent of the presence of the bacteria, which relieves us of the potential problems of antimicrobial or immunological clearance of the organism. (3) Extrapolation of the results obtained in RMs and rabbits for inhalation anthrax [49,50,51,60] to humans makes it possible to predict patient survival based on the level of these toxins, with a threshold beyond which antibiotic treatment is ineffective. The LF/EF ratio can be associated with the stage of the disease and PA is detectable at the intermediate stage of the disease using current techniques. Knowing the stage of the disease also allows the readjustment of treatment. Walsh et al. have shown that LF remains detectable in the blood for 12 days after antimicrobial therapy [52]. Antimicrobial therapy alone may not be sufficient if toxin levels are too high, as shown in the study of Boyer et al. with RMs [49] and as implied in the study of Weiner et al. [54], in which late debridement decreases the chances of survival of the host. 4) It makes it possible to monitor the effectiveness of treatment and seroconversion, either by directly measuring the toxins or by searching for anti-toxin antibodies, as applied in human cases of cutaneous, gastrointestinal, and inhalation anthrax [52,53]. Measuring toxin levels may help to monitor the efficiency of anti-toxin, as it is still the only specific authorized treatment to complement antibiotics [61].

## Figures and Tables

**Table 1:** Comparison of the direct assays for the detection of anthrax toxins.

Direct Method of Detection	Positive Points	Limit of Detection (LOD)	Negative Points	References
Ouchterlony method	Replaced in vivo passive protection and edema neutralization tests		Less sensitive and discriminative than indirect hemagglutination test (IHA) and enzyme-linked immunosorbent assay (ELISA) tests	[20,28]
ELISA PA, LF	Results within 2 h	LOD 1 ng/mL for protective antigen (PA) and 20 ng/mL for lethal factor (LF)	Late detection	[29]
ECLI, PA		LOD 1 ng/mL		[30]
ECLI, PA	Results in ≈ 35 min Sensitivity and specificity of 100% and 97%	LOD 2.5 ng/mL		[31]
Western-Blot PA, LF, EF			Interference of serum proteins Late detection	[32]
ENIA, PA	No interference by LF or edema factor (EF) Capacity to bind a high number of PA molecules	LOD 10 pg/mL	Mainly qualitative Concentration must be >1 µg/mL and <1 ng/mL for reliable results	[33]
MEF-PA assay	Results in 40 min	Sensitivity 1 pg/mL		[34]
SPR technology, PA		LOD 10 pg/mL		[35]
TRF, PA	Effective rate 90%	LOD 0.223 ng/mL for PA83 LOD 0.558 ng/mL for PA63	Interference of anthrax immune globulin (AIG) treatment Slight interference by LF binding for PA LOD	[36]
AlphaLISA, PA		LOD 100 pg/mL in spiked naïve sera LOD 2 ng/mL	PA spiked in serum	[37]
HTRF, PA	Assay in 15 min			[38]
LC-MS/MS, PA	Detection and quantification of total PA (PA83 + PA63) and PA83	Detection limits 1.3–2.9 ng/mL in plasma		[39]

**Table 2:** Comparison of assays using enzymatic activity for the detection of anthrax toxins.

Detection of Enzymatic Activity	Positive Points	LOD	Negative Points	References
MALDI-TOF MS, LF	No interference from PA83 or PA63	0.005–0.25 ng/mL	Late detection of LF	[42]
MALDI-TOF MS, LF		0.005–0.25 ng/mL	LOD varying according to the volume sample	[44]
MALDI-TOF MS, LT	Sensitivity and specificity of 100%	In plasma, detection limit of 0.033 ng/mL and 0.0075 ng/mL for the 2- and 1.8 h reaction times		[42,45]
LC-MS/MS, LF	Assay directly in the sample, without an immunocapture step	In the plasma, detection limit of 0.4 ng/mL	High detection limit in the ear mouse (40 ng/mL) Lower sensitivity and specificity	[46]
New PCR-based assay, LF	Rapid assay	Detection of 50 fg of LF spiked into human serum		[41]
MAPKKide based assay, LF	Rapid	After capture of LF, LOD< 5 pg/mL with HPLC LOD 20 pg/mL with microplate reader Directly in sample, LOD < 1 ng/mL in 5 h LOD 25 ng/mL in 15 min		[43]
ELISA, EF	Detection directly in sample Rapid (4 h)	LOD 1 pg/mL in human plasma LOD 10 pg/mL in animal plasma	Risk of false-positive with toxins of *Bordetella pertussis* and *Pseudomonas aeruginosa*	[47]
ELISA, EF	Detection directly in sample Rapid	LOD 2.5 pg/mL in mouse plasma LOD 0.85 pg/ear mouse tissue	Risk of false-positive with toxins of *Bordetella pertussis* and *Pseudomonas aeruginosa*	[46]
Monitoring ATP depletion, EF	Rapid (30 min)		Sensitivity of 0.1 μg/mL Addition of anti-EF antibodies	[48]
LC/ESI-MS/MS, EF		Detection limit 1000 times lower than that of LF		[44]
MALDI-TOF MS, total EF, ETx		In the plasma, LOD of 0.02 pg/mL for EF and ETx		[49,50]
LC-MS/MS, EF	Sensitivity and specificity of 100%	In the plasma, detection limit of 20 fg/mL		[51]
LC-MS/MS, total PA (PA83 + PA63) and PA83		Detection limits 1.3–2.9 ng/mL in 100 μL plasma		[39]
MALDI-TOF MS, total PA, PA83		LOD of 1.87 ng/mL for total PA and 1.22 ng/mL for PA83		[49,50]

**Table 3:** First detection of PA, LF, and EF in humans with cutaneous anthrax and in animal models of cutaneous anthrax (*BLI: bioluminescence).

Method of Detection	Model or Cases of Infection	First Time of Detection	Level of Toxins	References
MS	Human	Three to eight days after onset of symptoms	0.0005 < LF < 1.264 ng/mL, serum	[40]
TRF for PA MALDI-TOF MS for LF	Human	One to eight days after onset of symptoms	1.02 < PA < 68.73 ng/mL, serum 0.035 < LF < 1.264 ng/mL, serum	[36]
Western blot	Rabbit	48 h	3.6 < PA < 49.4 μg/mL 10.3 < LF < 35.2 μg/mL 1.9 < EF < 6.1 μg/mL	[32]
MALDI-TOF MS	Mouse	12 h	458 pg LF/injected ear 28 pg LF/cLN 476 pg LF/mL serum	[54]
MALDI-TOF MS	Mouse	Early phase of infection defined as BLI* in injected ear	16.25 ng LF/g of ear 0.253 ng LF/g of cLN 0.894 ng LF/mL serum LF detected in heart, lungs, spleen, liver	[55]
LC-MS/MS for LF EIA for EF	Mouse	Thirty minutes to 3 h 30 min	198 ng LF/ear 1.2 pg EF/ear 1.7 ng LF/mL plasma 4.6 pg EF/mL plasma	[46]

**Table 4:** First detection of PA, LF, and EF in humans with inhalation anthrax and in animal models of inhalation anthrax.

Method of Detection	Model or Cases of Infection	First Time of Detection	Level of Toxins	References
MS	Human	Three to four days after onset of symptoms	LF, 294.3 ng/mL, plasma	[52]
TRF for PA MALDI-TOF MS for LF	Human	Two and eight days after onset of symptoms	PA, 1.81 & 68.73 ng/mL, serum LF, 0.7 & 57.9 ng/mL, serum	[36]
MALDI-TOF MS	Human	A few days after onset of symptoms Initial drainage	LF, 58 ng/mL, plasma LF, 16.2 ng/mL, pleural fluid	[53]
ELISA	Guinea pig Rabbit	72–81 h (before death) ≈ 48 h	0.1 < PA < 1.7 μg/mL, serum 80 < PA < 100 μg/mL, serum 11 < LF < 15 μg/mL, serum	[29]
ELISA, ECLI	Guinea pig Rabbit	24 h 18 h	PA, ≈ 2 ng/mL 1 < PA < 10 ng/mL, serum	[30]
MALDI-TOF MS	RM	Two days post-infection	30 < PA <2 50 ng/mL, serum	[42]
MALDI-TOF MS	RM	24 h	0.006 < LF < 0.2 ng/mL, serum, 60% of animals	[58]
MALDI-TOF MS	RM	18 h for the first, 24 h for the second	Total LF, 0.026 ng/mL, serum Total LF, 0.049 ng/mL, serum	[45]
MS	Rabbits	12 h for LF 24 h for EF		[50]
LC-MS/MS	RM	24 h	EF, 0.16 and 0.462 pg/mL, serum, 40% of RM	[51]
LC-MS/MS	RM	48 h	84.3 < PA63 < 310 ng/mL, serum, 100% of RM	[39]
LC-MS/MS for LF EIA for EF	Mouse	1 h	LF, 2.63 ng/mL, plasma, all mice EF, 5.5 pg/mL, plasma, 42% of mice	[57]

## References

[1] Moayeri M., Leppla S.H., Vrentas C., Pomerantsev A.P., Liu S. (2015). Anthrax Pathogenesis. Annu. Rev. Microbiol..

[2] Jernigan J.A., Stephens D.S., Ashford D.A., Omenaca C., Topiel M.S., Galbraith M., Tapper M., Fisk T.L., Zaki S., Popovic T. (2001). Bioterrorism-related inhalational anthrax: The first 10 cases reported in the United States. Emerg. Infect. Dis..

[3] WHO Anthrax in Humans and Animals. https://apps.who.int/iris/bitstream/handle/10665/97503/9789241547536_eng.pdf;jsessionid=3BF3DA0CD1CA21CFBCF1160F6B7FB128?sequence=1.

[4] Antwerpen M.H., Zimmermann P., Bewley K., Frangoulidis D., Meyer H. (2008). Real-time PCR system targeting a chromosomal marker specific for Bacillus anthracis. Mol. Cell. Probes.

[5] van der Goot G., Young J.A. (2009). Receptors of anthrax toxin and cell entry. Mol. Asp. Med..

[6] Martchenko M., Jeong S.Y., Cohen S.N. (2010). Heterodimeric integrin complexes containing beta1-integrin promote internalization and lethality of anthrax toxin. Proc. Natl. Acad. Sci. USA..

[7] Molloy S.S., Bresnahan P.A., Leppla S.H., Klimpel K.R., Thomas G. (1992). Human furin is a calcium-dependent serine endoprotease that recognizes the sequence Arg-X-X-Arg and efficiently cleaves anthrax toxin protective antigen. J. Biol. Chem..

[8] Friebe S., van der Goot F.G., Burgi J. (2016). The Ins and Outs of Anthrax Toxin. Toxins.

[9] Vitale G., Pellizzari R., Recchi C., Napolitani G., Mock M., Montecucco C. (1998). Anthrax lethal factor cleaves the N-terminus of MAPKKs and induces tyrosine/threonine phosphorylation of MAPKs in cultured macrophages. J. Appl. Microbiol..

[10] Hellmich K.A., Levinsohn J.L., Fattah R., Newman Z.L., Maier N., Sastalla I., Liu S., Leppla S.H., Moayeri M. (2012). Anthrax lethal factor cleaves mouse nlrp1b in both toxin-sensitive and toxin-resistant macrophages. PLoS ONE.

[11] Chavarria-Smith J., Vance R.E. (2013). Direct proteolytic cleavage of NLRP1B is necessary and sufficient for inflammasome activation by anthrax lethal factor. PLoS Pathog..

[12] Leppla S.H. (1982). Anthrax toxin edema factor: A bacterial adenylate cyclase that increases cyclic AMP concentrations of eukaryotic cells. Proce. Natl. Acad. Sci. USA..

[13] Puhar A., Dal Molin F., Horvath S., Ladant D., Montecucco C. (2008). Anthrax edema toxin modulates PKA- and CREB-dependent signaling in two phases. PLoS ONE.

[14] Hong J., Doebele R.C., Lingen M.W., Quilliam L.A., Tang W.J., Rosner M.R. (2007). Anthrax edema toxin inhibits endothelial cell chemotaxis via Epac and Rap1. J. Biol. Chem..

[15] Smith H., Keppie J. (1954). Observations on experimental anthrax; demonstration of a specific lethal factor produced in vivo by Bacillus anthracis. Nature.

[16] Smith H., Keppie J., Stanley J.L. (1954). Observations on the cause of death in experimental anthrax. Lancet.

[17] Smith H., Tempest D.W., Stanley J.L., Harris-Smith P.W., Gallop R.C. (1956). The chemical basis of the virulence of Bacillus anthracis. *VII.* Two components of the anthrax toxin: Their relationship to known immunising aggressins. Br. J. Exp. Pathol..

[18] Strange R.E., Thorne C.B. (1958). Further purification studies on the protective antigen of Bacillus anthracis produced in vitro. J. Bact..

[19] Beall F.A., Taylor M.J., Thorne C.B. (1962). Rapid lethal effect in rats of a third component found upon fractionating the toxin of Bacillus anthracis. J. Bacteriol..

[20] Thorne C.B., Belton F.C. (1957). An agar-diffusion method for titrating Bacillus anthracis immunizing antigen and its application to a study of antigen production. J. Gen. Microb..

[21] Sargeant K., Stanley J.L., Smith H. (1960). The serological relationship between purified preparations of factors I and II of the anthrax toxin produced in vivo and in vitro. J. Gen. Microb..

[22] Stanley J.L., Sargeant K., Smith H. (1960). Purification of factors I and II of the anthrax toxin produced in vivo. J. Gen. Microbiol..

[23] Norman P.S., Ray J.G., Brachman P.S., Plotkin S.A., Pagano J.S. (1960). Serologic testing for anthrax antibodies in workers in a goat hair processing mill. Am. J. Hyg..

[24] Plotkin S.A., Brachman P.S., Utell M., Bumford F.H., Atchison M.M. (1960). An epidemic of inhalation anthrax, the first in the twentieth century. I. Clin. Features. Am. J. Med..

[25] Brachman P.S., Plotkin S.A., Bumford F.H., Atchison M.M. (1960). An epidemic of inhalation anthrax: The first in the twentieth century. Ii. Epidemiol. Am. J. Hyg..

[26] Darlow H.M., Pride N.B. (1969). Serological diagnosis of anthrax. Lancet.

[27] Fish D.C., Lincoln R.E. (1968). In vivo-produced anthrax toxin. J. Bacteriol..

[28] Turnbull P.C., Broster M.G., Carman J.A., Manchee R.J., Melling J. (1986). Development of antibodies to protective antigen and lethal factor components of anthrax toxin in humans and guinea pigs and their relevance to protective immunity. Infect. Immun..

[29] Mabry R., Brasky K., Geiger R., Carrion R., Hubbard G.B., Leppla S., Patterson J.L., Georgiou G., Iverson B.L. (2006). Detection of anthrax toxin in the serum of animals infected with Bacillus anthracis by using engineered immunoassays. Clin. Vaccine Immunol..

[30] Kobiler D., Weiss S., Levy H., Fisher M., Mechaly A., Pass A., Altboum Z. (2006). Protective antigen as a correlative marker for anthrax in animal models. Infect. Immun..

[31] Rossi C.A., Ulrich M., Norris S., Reed D.S., Pitt L.M., Leffel E.K. (2008). Identification of a surrogate marker for infection in the African green monkey model of inhalation anthrax. Infect. Immun..

[32] Molin F.D., Fasanella A., Simonato M., Garofolo G., Montecucco C., Tonello F. (2008). Ratio of lethal and edema factors in rabbit systemic anthrax. Toxicon.

[33] Tang S., Moayeri M., Chen Z., Harma H., Zhao J., Hu H., Purcell R.H., Leppla S.H., Hewlett I.K. (2009). Detection of anthrax toxin by an ultrasensitive immunoassay using europium nanoparticles. Clin. Vaccine Immunol..

[34] Dragan A.I., Albrecht M.T., Pavlovic R., Keane-Myers A.M., Geddes C.D. (2012). Ultra-fast pg/mL anthrax toxin (protective antigen) detection assay based on microwave-accelerated metal-enhanced fluorescence. Anal. Biochem..

[35] Ghosh N., Gupta N., Gupta G., Boopathi M., Pal V., Goel A.K. (2013). Detection of protective antigen, an anthrax specific toxin in human serum by using surface plasmon resonance. Diagn. Microbiol. Infect. Dis..

[36] Stoddard R.A., Quinn C.P., Schiffer J.M., Boyer A.E., Goldstein J., Bagarozzi D.A., Soroka S.D., Dauphin L.A., Hoffmaster A.R. (2014). Detection of anthrax protective antigen (PA) using europium labeled anti-PA monoclonal antibody and time-resolved fluorescence. J. Immunol. Methods.

[37] Mechaly A., Cohen N., Weiss S., Zahavy E. (2013). A novel homogeneous immunoassay for anthrax detection based on the AlphaLISA method: Detection of B. anthracis spores and protective antigen (PA) in complex samples. Anal. Bioanal. Chem..

[38] Cohen N., Mechaly A., Mazor O., Fisher M., Zahavy E. (2014). Rapid homogenous time-resolved fluorescence (HTRF) immunoassay for anthrax detection. J. Fluoresc..

[39] Solano M.I., Woolfitt A.R., Boyer A.E., Lins R.C., Isbell K., Gallegos-Candela M., Moura H., Pierce C.L., Barr J.R. (2019). Accurate and selective quantification of anthrax protective antigen in plasma by immunocapture and isotope dilution mass spectrometry. Analyst.

[40] Boyer A.E., Quinn C.P., Beesley C.A., Gallegos-Candela M., Marston C.K., Cronin L.X., Lins R.C., Stoddard R.A., Li H., Schiffer J. (2011). Lethal factor toxemia and anti-protective antigen antibody activity in naturally acquired cutaneous anthrax. J. Infect. Dis..

[41] Kolesnikov A.V., Kozyr A.V., Ryabko A.K., Shemyakin I.G. (2016). Ultrasensitive detection of protease activity of anthrax and botulinum toxins by a new PCR-based assay. Pathog. Dis..

[42] Boyer A.E., Quinn C.P., Woolfitt A.R., Pirkle J.L., McWilliams L.G., Stamey K.L., Bagarozzi D.A., Hart J.C., Barr J.R. (2007). Detection and quantification of anthrax lethal factor in serum by mass spectrometry. Anal. Chem..

[43] Suryadi K., Shine N. (2018). Design and use of a novel substrate for simple, rapid, and specific early detection of anthrax infection. PLoS ONE.

[44] Boyer A.E., Gallegos-Candela M., Lins R.C., Kuklenyik Z., Woolfitt A., Moura H., Kalb S., Quinn C.P., Barr J.R. (2011). Quantitative mass spectrometry for bacterial protein toxins—A sensitive, specific, high-throughput tool for detection and diagnosis. Molecules.

[45] Boyer A.E., Gallegos-Candela M., Quinn C.P., Woolfitt A.R., Brumlow J.O., Isbell K., Hoffmaster A.R., Lins R.C., Barr J.R. (2015). High-sensitivity MALDI-TOF MS quantification of anthrax lethal toxin for diagnostics and evaluation of medical countermeasures. Anal. Bioanal. Chem..

[46] Rougeaux C., Becher F., Ezan E., Tournier J.N., Goossens P.L. (2016). In vivo dynamics of active edema and lethal factors during anthrax. Sci. Rep..

[47] Duriez E., Goossens P.L., Becher F., Ezan E. (2009). Femtomolar detection of the anthrax edema factor in human and animal plasma. Anal. Chem..

[48] Israeli M., Rotem S., Elia U., Bar-Haim E., Cohen O., Chitlaru T. (2016). A Simple Luminescent Adenylate-Cyclase Functional Assay for Evaluation of Bacillus anthracis Edema Factor Activity. Toxins.

[49] Boyer A.E., Woolfitt A.R., Candela M., Lins R.C., Solano M., Lee J., Sanford D., Stark G., Dreier T., Quinn P. Toxin levels in organ tissues of nonhuman primates with inhalation anthrax. Proceedings of the International Conference on Bacillus anthracis, B. cereus and B. thuringiensis.

[50] Woolfitt A.R., Juni B.A., Gallegos-Candela M., Lins R., Solano M., Lee J., Sanford D., Stark G., Dreier T., Barr J. Development of anthrax toxemia in new zealand white rabbits developing systemic anthrax after exposure to low-dose ames spores. Proceedings of the International Conference on Bacillus anthracis, B. cereus, B. thuringiensis.

[51] Lins R.C., Boyer A.E., Kuklenyik Z., Woolfitt A.R., Goldstein J., Hoffmaster A.R., Gallegos-Candela M., Leysath C.E., Chen Z., Brumlow J.O. (2019). Zeptomole per milliliter detection and quantification of edema factor in plasma by LC-MS/MS yields insights into toxemia and the progression of inhalation anthrax. Anal. Bioanal. Chem..

[52] Walsh J.J., Pesik N., Quinn C.P., Urdaneta V., Dykewicz C.A., Boyer A.E., Guarner J., Wilkins P., Norville K.J., Barr J.R. (2007). A case of naturally acquired inhalation anthrax: Clinical care and analyses of anti-protective antigen immunoglobulin G and lethal factor. Clin. Infect. Dis..

[53] Sprenkle M.D., Griffith J., Marinelli W., Boyer A.E., Quinn C.P., Pesik N.T., Hoffmaster A., Keenan J., Juni B.A., Blaney D.D. (2014). Lethal factor and anti-protective antigen IgG levels associated with inhalation anthrax, Minnesota, USA. Emerg. Infect. Dis..

[54] Weiner Z.P., Boyer A.E., Gallegos-Candela M., Cardani A.N., Barr J.R., Glomski I.J. (2012). Debridement increases survival in a mouse model of subcutaneous anthrax. PLoS ONE.

[55] Weiner Z.P., Ernst S.M., Boyer A.E., Gallegos-Candela M., Barr J.R., Glomski I.J. (2014). Circulating lethal toxin decreases the ability of neutrophils to respond to Bacillus anthracis. Cell Microbiol.

[56] Marston C.K., Ibrahim H., Lee P., Churchwell G., Gumke M., Stanek D., Gee J.E., Boyer A.E., Gallegos-Candela M., Barr J.R. (2016). Anthrax Toxin-Expressing Bacillus cereus Isolated from an Anthrax-Like Eschar. PLoS ONE.

[57] Rougeaux C., Becher F., Goossens P.L., Tournier J.N. (2020). Very Early Blood Diffusion of the Active Lethal and Edema Factors of Bacillus anthracis After Intranasal Infection. J. Infect. Dis..

[58] Boyer A.E., Quinn C.P., Hoffmaster A.R., Kozel T.R., Saile E., Marston C.K., Percival A., Plikaytis B.D., Woolfitt A.R., Gallegos M. (2009). Kinetics of lethal factor and poly-D-glutamic acid antigenemia during inhalation anthrax in rhesus macaques. Infect. Immun..

[59] Holty J.E., Bravata D.M., Liu H., Olshen R.A., McDonald K.M., Owens D.K. (2006). Systematic review: A century of inhalational anthrax cases from 1900 to 2005. Ann. Intern. Med..

[60] Gallegos-Candela M., Boyer A.E., Woolfitt A.R., Brumlow J., Lins R.C., Quinn C.P., Hoffmaster A.R., Meister G., Barr J.R. (2018). Validated MALDI-TOF-MS method for anthrax lethal factor provides early diagnosis and evaluation of therapeutics. Anal. Biochem..

[61] Tournier J.N., Rougeaux C., Biot F.V., Goossens P.L. (2019). Questionable Efficacy of Therapeutic Antibodies in the Treatment of Anthrax. mSphere.

